# Supplementary material for: NDV-3A vaccination prevents C. albicans colonization of jugular vein catheters in mice
Source: Sci Rep. 2019 Apr 17;9:6194. doi: 10.1038/s41598-019-42517-y (PMC6470131; doi:10.1038/s41598-019-42517-y)
Supplement: Supplementary file 1 — Supplementary Figure 1 [file 41598_2019_42517_MOESM1_ESM.pdf]

NDV-3A vaccination prevents *C. albicans* colonization of jugular vein catheters in mice

Abdullah Alqarihi, Shakti Singh, John E. Edwards, Jr., Ashraf S. Ibrahim, Priya Uppuluri

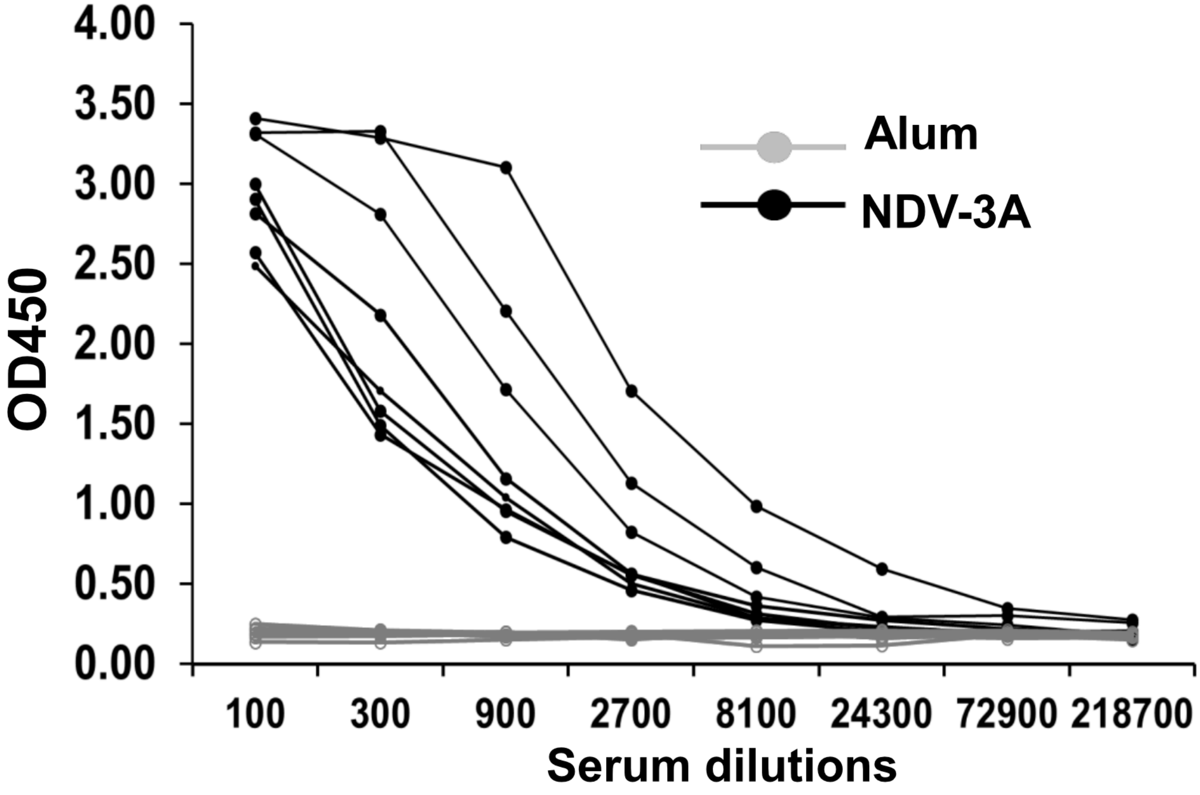

S1. Anti-NDV-3A vaccinated mice sera (n= 8 per group) displayed significantly high anti-rAls3p-N antibody titers compared to alum treated mice, even at dilutions as high as 1:2000.
